# *Staphylococcus aureus* ST1 promotes persistent urinary tract infection by highly expressing the urease

**DOI:** 10.3389/fmicb.2023.1101754

**Published:** 2023-02-22

**Authors:** Kai Xu, Yanan Wang, Ying Jian, Tianchi Chen, Qian Liu, Hua Wang, Min Li, Lei He

**Affiliations:** ^1^Department of Laboratory Medicine, Ren Ji Hospital, Shanghai Jiao Tong University School of Medicine, Shanghai, China; ^2^Faculty of Medical Laboratory Science, Shanghai Jiao Tong University School of Medicine, Shanghai, China

**Keywords:** *Staphylococcus aureus*, ST1, urease, persistence, urinary tract infection

## Abstract

*Staphylococcus aureus* (SA) is a relatively uncommon cause of urinary tract infections (UTIs) in the general population. Although rare, *S. aureus-*induced UTIs are prone to potentially life-threatening invasive infections such as bacteremia. To investigate the molecular epidemiology, phenotypic characteristics, and pathophysiology of *S. aureus-*induced UTIs, we analyzed non-repetitive 4,405 *S. aureus* isolates collected from various clinical sources from 2008 to 2020 from a general hospital in Shanghai, China. Among these, 193 isolates (4.38%) were cultivated from the midstream urine specimens. Epidemiological analysis showed UTI-derived ST1 (UTI-ST1) and UTI-ST5 are the primary sequence types of UTI-SA. Furthermore, we randomly selected 10 isolates from each of the UTI-ST1, non-UTI-ST1 (nUTI-ST1), and UTI-ST5 groups to characterize their *in vitro* and *in vivo* phenotypes. The *in vitro* phenotypic assays revealed that UTI-ST1 exhibits an obvious decline in hemolysis of human red blood cells and increased biofilm and adhesion in the urea-supplemented medium, compared to the medium without urea, while UTI-ST5 and nUTI-ST1 did not show significant differences between the biofilm-forming and adhesion abilities. In addition, the UTI-ST1 displayed intense urease activities by highly expressing urease genes, indicating the potential role of urease in UTI-ST1 survival and persistence. Furthermore, *in vitro* virulence assays using the UTI-ST1 *ureC* mutant showed no significant difference in the hemolytic and biofilm-forming phenotypes in the presence or absence of urea in the tryptic soy broth (TSB) medium. The *in vivo* UTI model also showed that the CFU of the UTI-ST1 *ureC* mutant rapidly reduced during UTI pathogenesis 72 h post-infection, while UTI-ST1 and UTI-ST5 persisted in the urine of the infected mice. Furthermore, the phenotypes and the urease expression of UTI-ST1 were found to be potentially regulated by the Agr system with the change in environmental pH. In summary, our results provide important insights into the role of urease in *S. aureus-*induced UTI pathogenesis in promoting bacterial persistence in the nutrient-limiting urinary microenvironment.

## Introduction

Urinary tract infections (UTIs) are common and recurrent infections that are often mild but can become life-threatening if left untreated. They are categorized as either uncomplicated or complicated and as either lower (bladder) or upper (pyelonephritis), based on their pathophysiology (Foxman, 2010). Although various species can cause UTI, most of the infections are caused by Gram-negative facultative anaerobic bacteria, such as *Escherichia coli*, *Klebsiella pneumoniae*, and *Proteus vulgaris*, and some infections are caused by Gram-positive bacteria, such as *Enterococcus faecalis*, *Clostridium perfringens*, and Staphylococci species (Donaldson, 1964; Hajdu et al., 2007; Foxman, 2010). *Staphylococcus aureus* (*S. aureus*, SA) may lead to complicated UTI in some diabetic, immunosuppressed, or catheter-applied patients (Zubair et al., 2019), while *Staphylococcus saprophyticus* (Flores-Mireles et al., 2015) may cause uncomplicated UTIs in sexually active females.

*Staphylococcus aureus*, a versatile and opportunistic pathogen, results in a wide range of infectious diseases, ranging from shallow skin infections to mortal endocarditis (Lowy, 1998). UTIs and Staphylococcal infections are common hospital-acquired infectious diseases (Ekkelenkamp et al., 2007; Hajdu et al., 2007; Foxman, 2010), although *S. aureus*-induced UTIs are uncommon, accounting for approximately 0.021%–1.53% of the UTIs (Ekkelenkamp et al., 2007; Foxman, 2010; Kitano et al., 2021). However, increased use of antibiotics has led to the emergence of methicillin-resistant *S. aureus* (MRSA) in the community and hospitals (Lee et al., 2018). Consequently, the incidence of MRSA-induced UTIs, which are resistant to routine antibiotic therapy, has increased in recent years, especially in immunocompromised or indwelling urinary catheter-applied patients (Karakonstantis and Kalemaki, 2018). Therefore, determining the molecular epidemiology, pathophysiology, and phenotypic characteristics of *S. aureus*-induced UTIs is necessary despite their relatively low prevalence.

Most Gram-negative bacteria initiate bladder infection through pili or biofilm formation by adhering directly to the tissues (Flores-Mireles et al., 2015). Moreover, a few uropathogens may secrete urease to survive the lower pH conditions in the bladder (Konieczna et al., 2012; Flores-Mireles et al., 2015; Zhou et al., 2019). Urease is the first metal-ion-containing enzyme to be isolated, and it is ubiquitous (Dixon et al., 1975; Konieczna et al., 2012). Many organisms, including plants, fungi, and bacteria, produce urease to hydrolyze urea, thus generating ammonia and carbonic acid (Krajewska and Ureases, 2009). Bacterial ureases show a wide range of compositions. Most bacterial ureases are composed of three subunits (α, β, and γ), nickel ions, and multiple accessory proteins, which are considered virulence factors (Rutherford, 2014). However, *Helicobacter pylori* urease lacks γ subunit but functions efficiently in elevating the pH of the stomach microenvironment (Konieczna et al., 2012). For urease-positive pathogens, urea hydrolysis can function as an acid response to elevate the acidic environment in the stomach or urine (Rutherford, 2014), thereby promoting epithelium destruction and renal stone formation (Rutherford, 2014; Schaffer et al., 2016). The majority of *S. aureus* (>90%) strains can produce urease; however, the clinical urease detection test—Christensen urea agar—is qualitative and time-consuming. Therefore, in this study, we used a modified semi-quantitative assay (Duran Ramirez et al., 2022) to measure the urease activity and the underlying pathogenic mechanism of *S. aureus*-induced UTI. Recently, there has been an increase in studies on Gram-positive pathogens and *S. aureus*-induced UTIs. Studies have revealed that urease is an essential factor in promoting bacterial colonization (Hiron et al., 2010; Remy et al., 2013; Duran Ramirez et al., 2022) and that copper resistance plays a role in MRSA-associated urinary tract fitness (Saenkham-Huntsinger et al., 2021).

For our study, we collected a total of 4,405 *S. aureus* isolates from various clinical sources during 2008–2020 from a general Hospital in Shanghai, China. We performed multilocus sequence typing (MLST) to identify the sequence type (ST) of the isolates and analyzed the antibiotic resistance profile to characterize the strains. In addition, we explored the potential pathogenesis of urinary tract infection-derived-sequence type (UTI-ST)-1, which is one of the main STs of UTI-derived SA (UTI-SA).

## Materials and methods

### Bacterial strains and growth conditions

For this study, we collected 4,405 non-repetitive *S. aureus* isolates from various clinical sources during 2008–2020 from a general Hospital in Shanghai, China. Thereafter, we randomly selected 10 isolates of UTI-ST1, UTI-ST5, nUTI-ST1, UTI-ST7, and UTI-ST398 to perform the phenotypic experiments. All the strains were grown in TSB (Oxoid, United States) in the presence or absence of urea supplementation (2% w/v; Yeasen, Shanghai, China). A nutrient-deficient medium, containing 1 g of peptone (Diamond, Shanghai, China), 1 g of D(+)-glucose (Sangon Biotech, Shanghai, China), 2 g of potassium dihydrogen phosphate (BBI, Shanghai), and 5 g of sodium chloride (Diamond, Shanghai) in 1 L of sterilized water, with or without 2% (w/v) urea-supplementation (Yeason), was prepared as described previously (Duran Ramirez et al., 2022), to conduct the urease activity test. The nutrient-deficient medium without phenol red was used to conduct the growth test.

### Growth curve

We selected 10 isolates of UTI-ST1 and UTI-ST5 for the growth curve test. The isolates were grown overnight in 3 mL TSB at 37°C and 220 rpm. The overnight cultures were then washed two times with phosphate-buffered saline (PBS) and diluted (1:10) in 3 mL fresh nutrient-deficient medium with or without urea supplementation (2% w/v). Thereafter, the diluted cultures were inoculated into sterile 96-well flat-bottom tissue culture plates (200 μL/well; Corning) and incubated at 37°C with shaking in Micro-ELISA Autoreader (Synergy 2, Bio-TeK, United States), and the OD was measured at 600 nm, every 30 min. The assay was performed in triplicate.

### Semi-quantitative biofilm assay

Semi-quantitative biofilm assay was performed as previously described (Vuong et al., 2003; He et al., 2019). Briefly, the overnight bacterial cultures were diluted with TSB, containing 0.5% glucose, to obtain a final OD of 0.05. Then, the diluted cultures were aliquoted to 96-well plates (200 μL/well) and incubated at 37°C for 24 h. The wells were washed with PBS after the gentle removal of the culture supernatants. Thereafter, the Bouin fixative was added to the wells to treat the biofilms for 1 h. The fixative was then gently aspirated and the wells were washed thrice with PBS, and then stained with 0.4% (w/v) crystal violet (Sangon). Biofilm formation was determined by measuring the OD at 570 nm using the Micro-ELISA autoreader.

### Hemolysis test

The hemolysis test was conducted as described previously (He et al., 2018; Ridder et al., 2021). Hemolytic activities were determined by incubating the overnight culture-supernatants with human red blood cells (RBCs; 2% v/v in Dulbecco’s phosphate-buffered saline, DPBS) for 1 h at 37°C and measuring the OD at 540 nm using an enzyme-linked immunosorbent assay (ELISA) reader. The assay was performed in triplicate. MRSA strain USA300 was used as a reference, as it displayed relatively strong virulence. A standard curve was made by using serial dilutions (1,2, 1:4, 1:8, and 1:16) of the supernatants of the RBC lysates.

### Adhesion and invasion of UTI-ST1 and nUTI-ST1 to human bladder epithelial 5,637 cells

The adhesion and invasion test was performed as described previously (Wang et al., 2018). The human bladder carcinoma 5,637 cells were cultured in Roswell Park Memorial Institute (RPMI) medium, supplemented with 10% fetal bovine serum (FBS) and 0.1% (w/v) urea, at 37°C and 5% CO_2_ and inoculated (2 × 10^5^ cells/well) in a 24-well plate. The UTI-ST1 and UTI-ST5 isolates were cultivated in TSB, with or without urea supplementation, to the mid-logarithmic growth phase and washed twice with PBS. Thereafter, the epithelial cells were infected with the UTI isolates at a 1:10 ratio. The adhesion assay was performed by co-incubating bacterial cells and epithelial cells for 2 h at 37°C and 5% CO_2_. The cells were washed three times with PBS to remove the planktonic bacteria and lysed with 0.1% sodium deoxycholate (Sangon) to release the adhered bacteria. Bacterial CFU was determined by serial dilutions of epithelial cell lysates on TSA plates. For the invasion test, the cells were incubated with bacteria for 4 h, and gentamicin (100 μg/mL) was added to the culture for 30 min to digest the bacteria outside the cells. Thereafter, the bacterial CFU was enumerated to assess the invasion ability.

### Urease activity

The nutrient-deficient medium with phenol red was used to perform the bacterial urease activity test. The overnight cultures of different isolates were centrifuged at 4,000 rpm and washed with PBS to remove the secreted urease. Bacteria were resuspended in the nutrient-deficient medium, with or without urea supplementation, and diluted to a final OD of 0.03–0.04. The urease activity was measured by continuously monitoring the OD at 560 and 415 nm, every 30 min, on the Micro-ELISA autoreader. *Proteus mirabilis* and *E. coli* were used as urease-positive and -negative controls, respectively.

### Quantitative reverse transcription PCR

The transcription of urease (*ureACD*) and accessory gene regulator (*agr*) effector (RNAIII) genes in UTI-ST1 and UTI-ST5 was detected by Quantitative reverse transcription PCR (RT-qPCR). Complementary DNA (cDNA) was synthesized from total RNA using the PrimeScript™ reverse transcriptase kit (Takara), according to the manufacturer’s instructions. Thereafter, the cDNA samples were amplified using the FastStart Universal SYBR Green Master kit (Roche). The reactions were performed on a 7500 Sequence Detector (Applied Biosystems). Purified chromosomal DNA (0.005–50 ng/mL) was used to construct a standard curve. The reactions were performed in triplicate, and DNA gyrase subunit B (*gyrB*) was used as an internal reference.

### MLST of *Staphylococcus aureus* isolates

MLST was performed using seven housekeeping genes (*arcC*, *areO*, *yqiL*, *glp*, *pta*, *tpi*, and *gmk*). The sequences of the PCR products were compared with the references on the MLST website^1^ for *S. aureus*.

### Murine UTI model

The murine UTI model was constructed as described previously (Hagberg et al., 1983; Armbruster et al., 2018; Rashid et al., 2021). Female C57BL/6 mice (aged 6–8 weeks, housed 5/cage) were obtained from JSJ Laboratory Animals LTD (Shanghai, China) and subjected to bacterial infection after 3 days, after overnight water-deprivation, to void the bladders in case of immediate micturition after transurethral infection. Subsequently, the bacterial culture (5 × 10^8^ CFU/100 μL) was transurethrally injected into the anesthetized mice with a catheter needle (28G). Each mouse was inoculated with one kind of bacterial culture. Urine was collected every 24 h by massaging the abdomen of the mice and 10 μL of urine was used for serial dilution to calculate bacterial CFU. Bladders and kidneys were harvested for the quantification of bacterial burden (CFU/mL) at 72 hpi, by serial dilution plating and colony enumeration of homogenized organs after 24-h incubation.

### Intracellular bacterial persistence in neutrophils and lactate dehydrogenase assay

#### Neutrophils isolation

To conduct the cytotoxicity detection test, the neutrophils were isolated from the venous blood of healthy individuals as described previously (Kremserova and Nauseef, 2020). The anticoagulant blood was mixed with 3% dextran and incubated for 20 min to separate the erythrocytes from the leukocytes. Thereafter, the neutrophils were isolated by a discontinuous density gradient of Ficoll–Hypaque (Sigma, Germany). After centrifugation at 400 g for 40 min at room temperature, the supernatant was discarded and the erythrocytes were lysed. Finally, the neutrophils were washed with PBS three times and suspended in RPMI (Hyclone, United States).

#### Intracellular bacterial persistence

The isolated neutrophils and bacteria were co-incubated at 1:100 for 2 or 5 h at 37°C and 5% CO_2_, in 96-well plates. The plates were centrifuged at 400 g for 10 min to remove the supernatant and each well was washed three times with PBS. The samples were serially diluted to calculate bacterial CFU, as described earlier.

#### LDH assay

The cytotoxicity was detected using the lactate dehydrogenase (LDH) cytotoxicity detection kit assay (Roche, Germany). For this assay, the isolated neutrophils and bacteria were co-incubated for 2 or 5h at a 1:100 ratio in RPMI. In addition, three cell samples without bacteria and three cell samples with 0.1% Triton X-100 were set as negative and positive controls, respectively. The OD of the samples at 490 or 540 nm was recorded.

#### pH measurement

The pH of the cultures was measured by using a pH meter (Leici, Shanghai, China) as described previously (Zhou et al., 2019). Tris–HCl solutions (pH = 8.0 and 6.0) were used as pH standard solutions for calibration.

#### Molecular genetic techniques

To construct the UTI-ST1 *ureC* and *agrA* mutants, a homologous recombination procedure was performed as described previously (Liu et al., 2015; Dai et al., 2017), using pKOR1 plasmid. The mutated DNA fragments were PCR-amplified from chromosomal DNA of *S. aureus* isolate UTI-ST1-15-68, using *ureC*-A, -B, -C, and -D and *agrA*-A, -B, -C, and -D primers. These products were cloned into pKOR1 using clonase reaction and *attB* sites, yielding pKOR1-*ureC* and pKOR1-*agrA* plasmids. Thereafter, the recombinant plasmids were transferred first to *S. aureus* RN4220 and then to *S. aureus* isolate UTI-ST1-15-68 *via* electroporation. Proper integration was verified by analytical PCR and DNA sequencing of the PCR-derived regions, using pKOR-Up-ApiI-F and pKOR-Dn-KpnI-R primers. The primers used for amplification are listed in Supplementary Table S1. For genetic complementation, the *ureC* gene was amplified with a pair of primers, *ureC*-SmaI-F, and *ureC*-BamHI-R, and the *agrA* gene was amplified with *agrA*-SmaI-F and *agrA*-BamHI-R, using genome DNA of UTI-ST1-15-68 as a template. The amplified DNA fragment was connected to the vector pos1 with T4 ligase to generate a complementation plasmid. Then, the plasmid was transduced into the UTI-ST1 *ureC* mutant and UTI-ST1 *agrA* mutant strains with ϕ85. The detailed procedures were described earlier.

### Ethics approval

Animal experiments were performed in ABSL2 facilities following the Guide for the Care and Use of Laboratory Animal Sciences (CALAS) and approved by the ethics committee of Renji Hospital, School of Medicine, Shanghai Jiaotong University, Shanghai, China (Approval Number: KY2021-225-B).

### Statistical analysis

All statistical tests were performed with GraphPad Prism 8.0 software. Percentage values were analyzed pairwise by the two-tailed chi-square test or Fisher’s exact test. For comparisons of two groups, paired and unpaired two-tailed Student’s *t*-tests were applied. Error bars in all graphs indicated the standard error of the mean (mean ± SEM), and a *p-*value of <0.05 was considered statistically significant.

## Results

### Molecular characteristics of the UTI-SA

**Figure 1:**
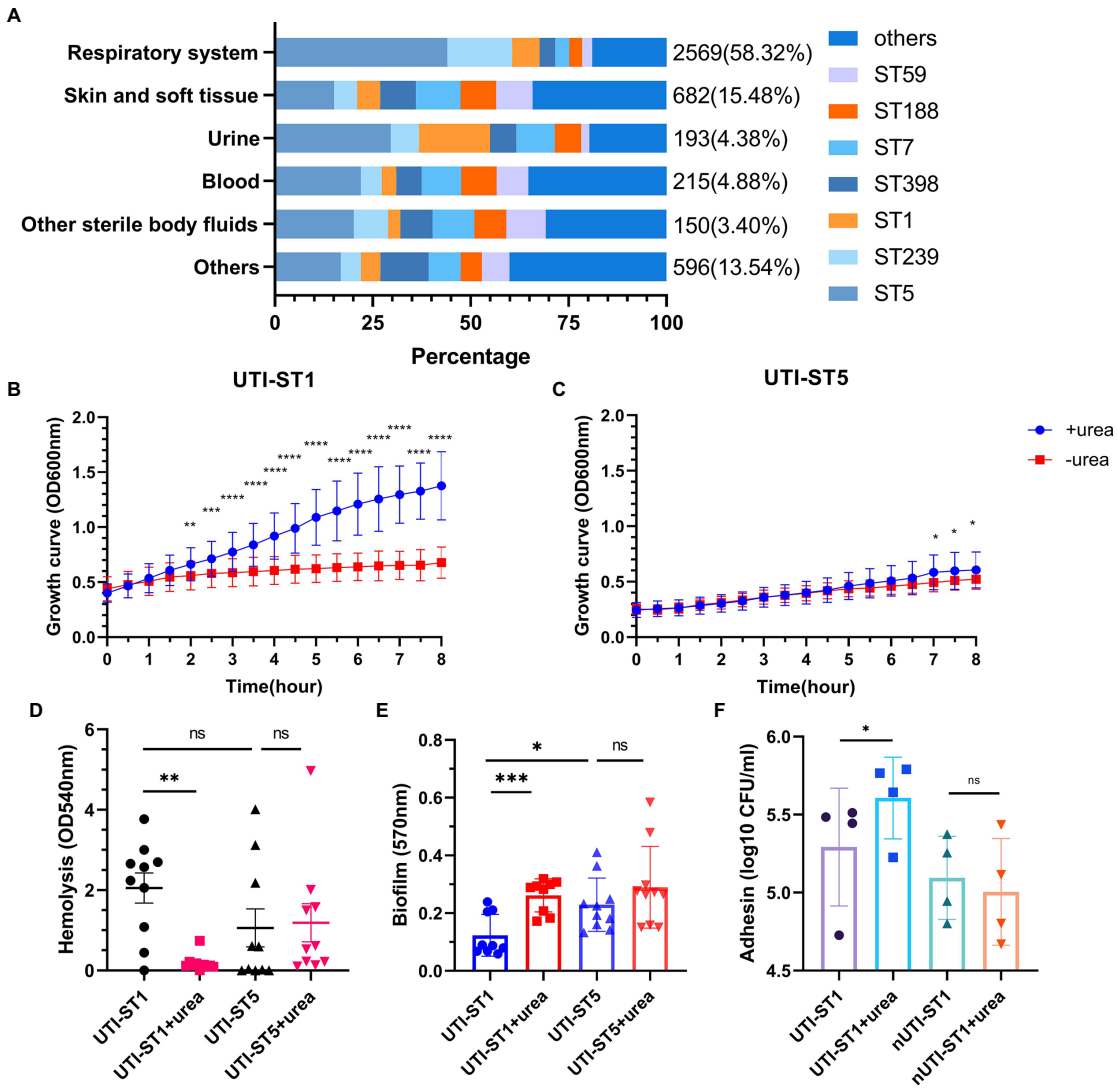
The proportion of sequence types (STs) in the *Staphylococcus aureus* samples and the phenotypic characteristics of urinary tract infection-derived-sequence type (UTI-ST)-1 and UTI-ST5 in the urea-supplemented and -unsupplemented media. **(A)** The proportion of UTI-ST5, UTI-ST239, UTI-ST1, UTI-ST398, UTI-ST7, UTI-ST188, and UTI-ST59 in different *S. aureus* samples; **(B,C)** 8-h growth curves of UTI-ST1 **(B)** and UTI-ST5 **(C)** in the presence or absence of 2% urea-supplementation in the modified nutrient-deficient medium at 37°C and 220 rpm, by continuous optical density (OD) measurement at 600 nm, every 30 min; **(D–F)** phenotypic experiments of UTI-ST1, UTI-ST5, and non-UTI-derived (nUTI)-ST1; analysis of the hemolytic activities of UTI-ST1 and UTI-ST5, in the presence or absence of 2% urea-supplementation, by measuring the OD values at 540 nm **(D)**; the biofilm-forming ability of UTI-ST1 and UTI-ST5, analyzed using semi-quantitative crystal violet staining by measuring OD at 570 nm in the presence or absence of 2% urea-supplementation **(E)**; the adhesion ability of UTI-ST1 and nUTI-ST1 to human bladder epithelium 5,637 cells **(F)**. The strains performed in the hemolysis, biofilm formation, and adhesion tests are listed in Supplementary Table S2. Each dot in the graphs of these three tests is an average of the triplicates of each strain. Statistical analyses were performed by using the Student’s *t*-test after the Shapiro–Wilk normality test, ^*^p < 0.05; ^**^p < 0.01; ^***^p < 0.001; ^****^p < 0.0001; ns, not significant.

In this prevalence survey, a total of 4,405 non-repetitive *S. aureus* strains were collected from various clinical sources during 2008–2020 from a general Hospital in Shanghai, China (Table 1). Among these strains, UTI-ST5 (33.37%) was the most prevalent ST, followed by UTI-ST239 (12.10%), which has shown a gradual decline in recent years (Dai et al., 2019), and UTI-ST1 (6.74%; Table 1). Screening of each ST by specimen type revealed that the midstream urine specimen isolations of UTI-ST1 (11.59%, *p* < 0.001) were significantly higher than the other STs (Table 1). In addition, among the 193 UTI-SA strains, the isolation rate of UTI-ST5 (29.53%) was the highest, followed by the isolation rates of UTI-ST1 (18.13%) and UTI-ST7 (9.84%; Figure 1A; Table 2). Furthermore, both UTI-ST1 and UTI-ST5 were prevalent in the elderly population (aged ≥65) compared to the young population (88.57% vs. 11.43%, *p* < 0.05 and 63.16% vs. 36.84%, *p* < 0.05, respectively), while UTI-ST7, UTI-ST398, and UTI-ST188 were more frequently found in the younger populations (aged < 65; Table 2) and UTI-ST239 showed a slight advantage in elderly people (Table 2). Moreover, the majority of UTI-ST1 (65.85%) and UTI-ST5 (81.67%) isolates were recovered from men (Supplementary Figure S1). Therefore, owing to the high prevalence and isolation rates of UTI-ST1 and UTI-ST5, these samples were selected to further characterize the UTI-SA strains.

**Table 1:** The sequence type proportion of total strains and the specimen sources distribution in each sequence type.

Sequence type (ST)	ST5	ST239	ST1	ST398	ST7	ST59	ST188	Others	Total strains
No. of total(%)	1,470(33.37%)	533(12.10%)	297(6.74%)	282(6.40%)	272(6.17%)	207(4.70%)	201(4.56%)	1,143(25.95%)	4,405(100%)
age (≥65/<65)(%)	46.44/53.56	47.23/52.77	70.14/29.86	27.96/72.04	36.90/63.10	27.77/75.23	24.10/75.90	36.95/63.05	42.06/57.94
**Specimen sources(%)**									
Respiratory system	77.01%	79.55%	60.61%	36.88%	33.45%	32.37%	41.79%	42.61%	58.32%
Skin/soft tissue	6.33%	6.75%	12.12%	19.86%	25.74%	27.54%	21.89%	19.34%	15.48%
Blood	2.70%	1.88%	2.36%	4.26%	6.99%	7.25%	8.45%	5.77%	4.88%
Urine	3.89%	2.63%	11.78%^***^	4.61%	6.99%	1.93%	5.47%	3.50%	4.38%
Sterile body fluids	2.18%	2.63%	1.68%	4.61%	6.25%	7.73%	3.98%	4.72%	3.40%
Others	7.82%	6.57%	11.45%	29.79%	20.59%	23.19%	18.41%	23.97%	13.54%

Statistical analysis was performed using the Chi-test, ^***^p < 0.001.

**Table 2:** The age antibiotic profile of popular UTI sequence types of *Staphylococcus aureus*.

Sequence type(ST)	No. of cases (percentage)	Age	Antibiotics resistance (%)/No.										
			PEN	GEN	CZO	ERY	FOS	RIF	SXT	LVX	LNZ	VAN	FOX
ST5	57(29.53%)	<65 (36.84%)	100.00/57	**84.21/48** ^***^	64.91/37	**85.96/49** ^***^	33.33/19	7.02/4	19.3/11	**63.16/36** ^**^	0.00/0	0.00/0	94.74/54
		≥65 (63.16%)											
ST1	35(18.13%)	<65 (11.43%)	97.14/34	8.57/3	45.71/16	11.43/4	25.71/9	0.00/0	2.86/1	91.43/32	0.00/0	0.00/0	91.43/32
		≥65 (**88.57%**)^*^											
ST7	19(9.84%)	<65 (64.71%)	100.00/17	29.41/5	0.00/0	**47.06/8** ^**^	0.00/0	0.00/0	5.88/1	**17.65/3** ^***^	0.00/0	0.00/0	**0.00/0** ^***^
		≥65 (**35.29%**)^***^											
ST239	14(7.25%)	<65 (42.86%)	**42.85/6** ^***^	28.57/4	21.43/3	35.71/5	21.43/3	7.14/1	0.00/0	**35.71/5** ^***^	0.00/0	0.00/0	**42.85/6** ^**^
		≥65 (**57.14%**)^*^											
ST398	13(6.74%)	<65 (76.92%)	**69.23/9** ^*^	15.38/2	**0.00/0** ^***^	**46.15/6** ^*^	7.69/1	15.38/2	0.00/0	**7.69/1** ^***^	0.00/0	0.00/0	**46.15/6** ^**^
		≥65 (**23.08%**)^***^											
ST188	11(5.70%)	<65 (54.55%)	90.91/10	9.09/1	**9.09/1** ^*^	**45.45/5** ^*^	0.00/0	0.00/0	0.00/0	**9.09/1** ^***^	0.00/0	0.00/0	**9.09/1** ^***^
		≥65 (**45.45%**)^*^											
Others	44(22.80%)	<65 (28.57%)	97.50/39	**32.50/13** ^*^	**15.00/6** ^*^	**60.00/24** ^***^	**2.50/1** ^*^	0.00/0	5.00/2	**37.50/15** ^***^	0.00/0	0.00/0	**52.50/21** ^***^
		≥65 (71.43%)											

Chi-test was used for statistical analysis, compared with UTI-ST1; PEN, penicillin; GEN, gentamicin; CZO, cefazolin; ERY, erythromycin; FOS, fosfomycin; RIF, rifampicin; SXT, sulfamethoxazole; LVX, levofloxacin; LVZ, linezolid; VAN, vancomycin; FOX, cefoxitin. The bold values mean the values are with significant differences compared with the other sequence types.

* *p* < 0.05.

** *p* < 0.01.

*** *p* < 0.001.

### Antibiotic resistance pattern among UTI-SA isolates

The antimicrobial pattern of UTI-ST1 was different from that of UTI-ST5 and other STs (Table 2). In general, UTI-ST5 and UTI-ST1 isolates were highly resistant to penicillin (100% and 97.14%, respectively) and cefoxitin (94.74% and 91.43%, respectively; Table 2). In contrast, gentamicin, erythromycin, and trimethoprim-sulfamethoxazole displayed potent antibiotic activity against UTI-ST1 (8.57%, 11.43%, and 2.86%, respectively), but not against UTI-ST5 (Table 2). In addition, among the STs, only UTI-ST1 exhibited resistance to levofloxacin (91.43%, *p* < 0.05; Table 2). The wide use of antibiotics in recent years has led to the generation of multiple antibiotic-resistance genes in bacteria, causing severe health problems. UTI-ST5 is a highly drug-resistant and less virulent ST, which is prevalent in the hospital, while UTI-ST398 and UTI-ST188 are community-associated STs with hypervirulence and low drug resistance. UTI-ST5 showed high drug resistance rates against most antibiotics, while UTI-ST1 displayed lower resistance to antibiotics, except for levofloxacin. Nitrofurantoin, trimethoprim-sulfamethoxazole, fosfomycin, and pivmecillinam are recommended for first-line therapy (Gupta et al., 2011) in UTI treatment, and β-lactams and fluoroquinolones are used as alternative treatments (Gupta et al., 2011, 2017), due to the prevalence of high antibiotic-resistance. This suggests that fluoroquinolone resistance in UTI-SA isolates is a major concern in UTI treatment.

### Urea supplement promotes UTI-ST1 to survive and persist in the urinary tract

The urinary tract is a urine-discharging system and human urine primarily consists of water (95%) and urea (2%; Sarigul et al., 2019; Volpin et al., 2019). The liver produces urea *via* the urea cycle, which is then released into the blood (Ha et al., 1947; Morris, 2002). The kidneys then filter the blood and concentrate urea and waste metabolites in the urine (Morris, 2002; Weiner et al., 2015). A previous study reported that urea supplementation in tryptic soy broth (TSB) supplemented with excess glucose, could reduce bacterial cell death during the stationary growth phase, due to ammonia generation and reduced intracellular reactive oxygen species levels (Zhou et al., 2019). To determine how UTI-ST1 and UTI-ST5 adapt to the high-urea environment of the bladder, we randomly selected 10 isolates from each group to test their growth, hemolytic ability, biofilm formation, and adhesion.

To investigate their growth competence, we conducted the growth assay in a modified nutrient-deficient medium, with or without urea supplementation (2%), at 37°C and 220 rpm. During the 8-h monitoring, the optical density (OD) at 600 nm was recorded every 30 min. The UTI-ST1 isolates grew significantly faster in the urea-supplemented medium than in the urea-unsupplemented medium (Figure 1B). In contrast, UTI-ST5 isolates did not show growth improvement with urea supplementation until 6-h growth (Figure 1C). These results suggest that UTI-ST1 can utilize urea to promote survival and growth, while UTI-ST5 displays a delayed utilization of urea. Furthermore, to assess the effect of urea supplementation on the pathogenicity of UTI-ST1 and UTI-ST5, we performed hemolysis and biofilm formation tests in TSB, with or without urea supplementation. After 10–12 h of cultivation, the supernatants of UTI-ST1 and UTI-ST5 from the urea-supplemented and -unsupplemented media were collected and incubated with erythrocytes (Ridder et al., 2021). Our results revealed that the cytolytic abilities of the UTI-ST1 isolates were highly weakened in the presence of urea (Figure 1D), while the hemolytic abilities of the UTI-ST5 isolates were not impacted by urea supplementation (Figure 1D). Similarly, UTI-ST1 biofilm was thicker in the urea-supplemented media, compared to that in the unsupplemented media (Figure 1E), while no significant difference was observed in the biofilms of the UTI-ST5 isolates, in the presence or absence of urea supplementation (Figure 1E). Since the UTI-ST5 strain is characterized by aggressive biofilm formation and hypovirulence (Figures 1D,E), we compared the hemolytic and biofilm-forming abilities of the non-UTI-derived (nUTI)-ST1 strains, which showed no significant differences in the presence and absence of urea supplementation (Supplementary Figures S2A,B). These results demonstrate that UTI-ST1 shows reduced virulence and thicker biofilm formation on polystyrene surfaces, such as an implant in the urea environment. However, the weakened virulence, although may cause less severe inflammatory reactions (Gong et al., 2014), biofilm formation in the urinary tract can reduce the flow of urine and antimicrobial agents (Scotland et al., 2019), facilitating persistent infection. Considering the elevated adhesion of UTI-ST1 to polystyrene in the urea-supplemented medium, we hypothesized that the adhesion of UTI-ST1 to the urinary epithelium could be improved by urea supplementation. Thus, 0.1% (w/w) urea was supplied to the cell medium to mimic the bladder microenvironment and it was observed that the UTI-ST1 isolates displayed stronger adhesion to the bladder epithelium 5,637 cells (Figure 1F), although their invasion ability was not improved (Supplementary Figure S2C).

We found that UTI-ST1 may shift to hypo virulent status and display increased biofilm formation on polystyrene, while the increased adhesion to epithelium helps UTI-ST1 to colonize and persist in the urinary tract. In addition, we hypothesized that UTI-ST5 may use its biofilm formation ability to survive in the urinary tract.

### Urease plays an important role in UTI-ST1 survival and persistence

**Figure 2:**
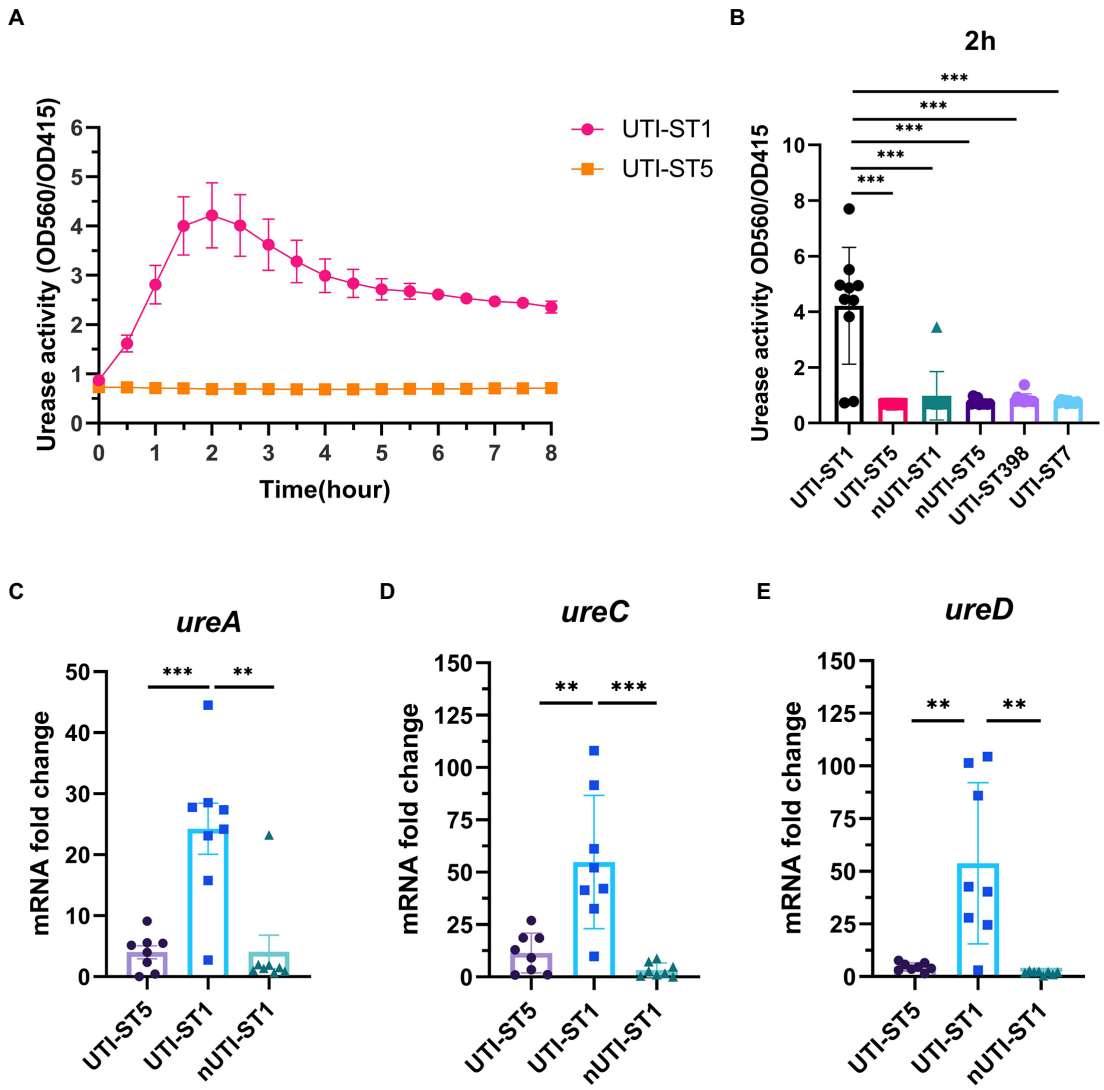
Urease activities of urinary tract infection-derived-sequence type (UTI-ST)-1 and UTI-ST5 and urease gene transcription in UTI-ST1, UTI-ST5, and non-UTI-derived (nUTI)-ST1. **(A,B)** Urease activities of UTI-ST1 and UTI-ST5, determined by measuring optical density (OD) at 560 and 415 nm, represented by the red and yellow curves, respectively **(A)**; OD was measured continuously for 2 h to quantitatively analyze the urease activities of UTI-ST1 and UTI-ST5 **(B)**. **(C–E)** The transcriptional level of urease genes (*ureA*, *ureC*, and *ureD*) was measured by quantitative reverse-transcription PCR (RT-qPCR). 10 strains from each sequence type were performed in urease activities; 8 isolates were used in urease gene transcription. The strains used were listed in Supplementary Table S2. Each dot in RT-qPCR is an average of the triplicates of each strain. The primers used in RT-qPCR are listed in Supplementary Table 1. The transcription level of *gyrB* was used for normalization. Unpaired Student’s *t*-test was used for statistical analyses, ^*^p < 0.05; ^**^p < 0.01; ^***^p < 0.001; ns, not significant.

Based on the previous results, we hypothesized that UTI-ST1 and UTI-ST5 may have disparate urease activities. However, the conventional urease detection assay—Christensen urea agar—is qualitative and has limited sensitivity. Therefore, in this study, we used a modified semi-quantitative method to evaluate the urease activity precisely and easily (Roberts et al., 1978; Duran Ramirez et al., 2022). We inoculated diluted overnight culture in a modified urea-supplemented (2%, w/v) medium (pH 6.5) containing phenol red, a pH indicator, which would turn red under alkaline conditions, such as when ammonia is generated. The OD at 560 and 415 nm was recorded to monitor the color change of the solution and the results revealed that the urease activity of UTI-ST1 reached its peak at 2 h (Figure 2A), while all the selected ST5 strains, albeit harboring the wwurease operon confirmed by DNA sequencing (data not shown) and other strains did not show any obvious change in the urease activities (Figure 2A; Supplementary Figure S3). Thereafter, we quantified the urease activities of common UTI and nUTI strains at 2 h (Figure 2B) and found that UTI-ST1 and UTI-ST5 strains varied significantly in their urea-utilizing ability.

**Figure 3:**
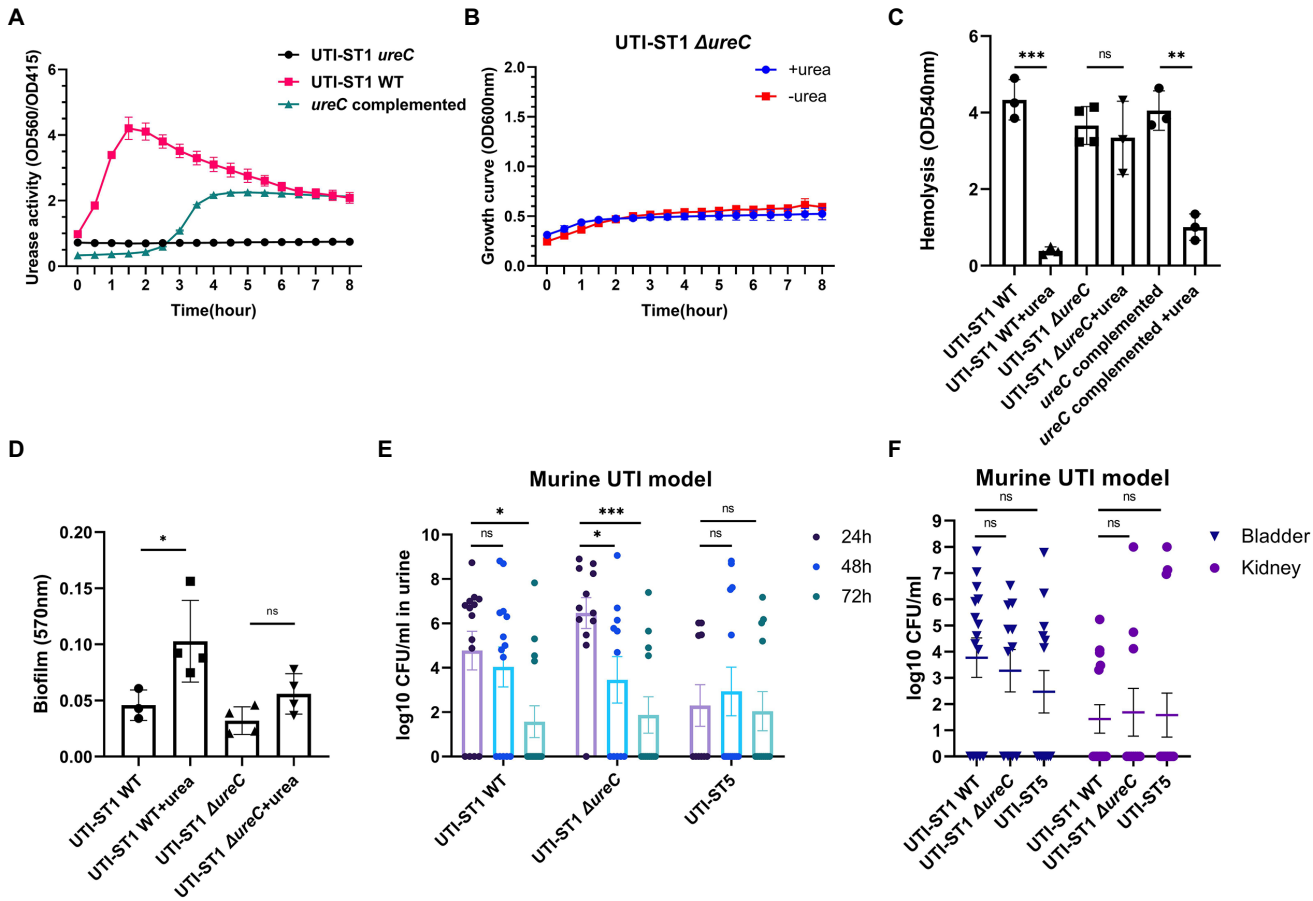
Phenotypic differences between urinary tract infection-derived-sequence type (UTI-ST)-1 wildtype (WT), UTI-ST1 *ureC* mutant, and *ureC* complemented either in the presence or absence of 2% urea supplementation and murine UTI model. **(A)** The urease activities of UTI-ST1 WT, UTI-ST1 *ureC* mutant and *ureC* complemented strain were determined by measuring optical density (OD) at 560 and 415 nm; growth curves **(B)**, hemolytic abilities **(C)**, and biofilm-forming abilities **(D)** of UTI-ST1 WT and UTI-ST1 *ureC* mutant; **(E,F)** 15 female C57BL/6 mice were infected with UTI-ST1 WT, UTI-ST1 *ureC* mutant or UTI-ST5 for each group were used to construct murine UTI model; urine was collected every 24 h **(E)**, and bladders and kidneys were harvested at 72 h and homogenized to calculate the CFU/mL **(F)**. Statistical analyses were performed using unpaired Student’s *t*-test, ^*^p < 0.05; ^**^p < 0.01; ^***^p < 0.001; ns, not significant.

Thus, to determine the cause of the differences in the urease activities of the *S. aureus* strains, we selected 2 strains of UTI-ST1 and nUTI-ST1 each, for sequencing analysis. However, we found no variations in the sequences (mutations, insertions, or deletions) of urease genes between the two strains (data not shown). Therefore, we hypothesized that the variations in urease activities of the *S. aureus* strains may arise at the transcriptional level. *S. aureus* urease is a complex nickel-containing enzyme (Krajewska and Ureases, 2009). It consists of three subunits, α, β, and γ, which are encoded by *UreC*, *ureB*, and *ureA*, respectively, and coenzymes encoded by *ureDEFH* (Krajewska and Ureases, 2009; Konieczna et al., 2012). The results of quantitative reverse transcription PCR (RT-qPCR) revealed that the transcription of *ureA, ureC, and ureD* was significantly higher in UTI-ST1, compared to that in UTI-ST5, and nUTI-ST1 (Figures 2C–E), indicating the positive urease activities of UTI-ST1. Since the α-subunit, encoded by *ureC*, is the active site of the urease enzyme, we constructed the UTI-ST1 *ureC* mutant and a complemented strain to confirm the function of urease in ST1-induce UTI. The results revealed that the UTI-ST1 *ureC* mutant lost the urease activity (Figure 3A) and growth improvement in the urea environment (Figure 3B), indicating that the mutant could not utilize urea. However, the mutant showed no significant difference in the hemolytic and biofilm-forming phenotypes in the presence or absence of urea in the TSB medium (Figures 3C,D). While, the complemented strain recovered the urease activities (Figure 3A) and exhibited a similar hemolysis phenotype change as the WT strain (Figure 3C), confirming the function of urease. In addition, to verify the function of urease *in vivo*, we constructed a murine UTI model, by transurethrally injecting the same inoculum amount of UTI-ST1 WT, UTI-ST1 *ureC* mutant, and UTI-ST5 (Rashid et al., 2021). On the first day post-infection (24 hpi), both UTI-ST1 WT and UTI-ST1 *ureC* mutants were successfully established in most of the mice, with the mutant group displaying higher bacterial burden in the urine (Supplementary Figure S4), although the difference was not statistically significant. This was possibly because the mutant strain showed stronger virulence compared to the WT, leading to an intense immune response and discharge of more bacterial particles. Therefore, the CFU of the urine from the UTI-ST5-infected mice was slightly lower and relatively stable than that of the urine from the UTI-ST1-infected mice (Figure 3E; Supplementary Figure S4), which was consistent with its high isolation rate compared to all the STs. At 48 hpi, the bacterial burden of the *ureC* mutant-infected group declined rapidly (~10-fold) compared to that of the WT-infected group (Figure 3E), confirming the increased adhesion results that were obtained earlier. While at 72 hpi, the CFUs in the urine of UIT-ST1-WT dropped significantly probably because of the reduced virulence in urine for UTI-ST1 leading to phenotype like UTI-ST5. The bacterial burden of the bladder and kidney at 72 hpi suggested that the UTI-ST1 WT and UTI-ST1 *ureC* mutant mainly focused on the lower urinary tract, probably leading to cystitis (Figure 3F). However, UTI-ST5 displayed kidney colonization, suggesting that UTI-ST5 can cause ascending infections, such as pyelonephritis or bloodstream infection.

### Urease gene expression changes with the environmental pH

**Figure 4:**
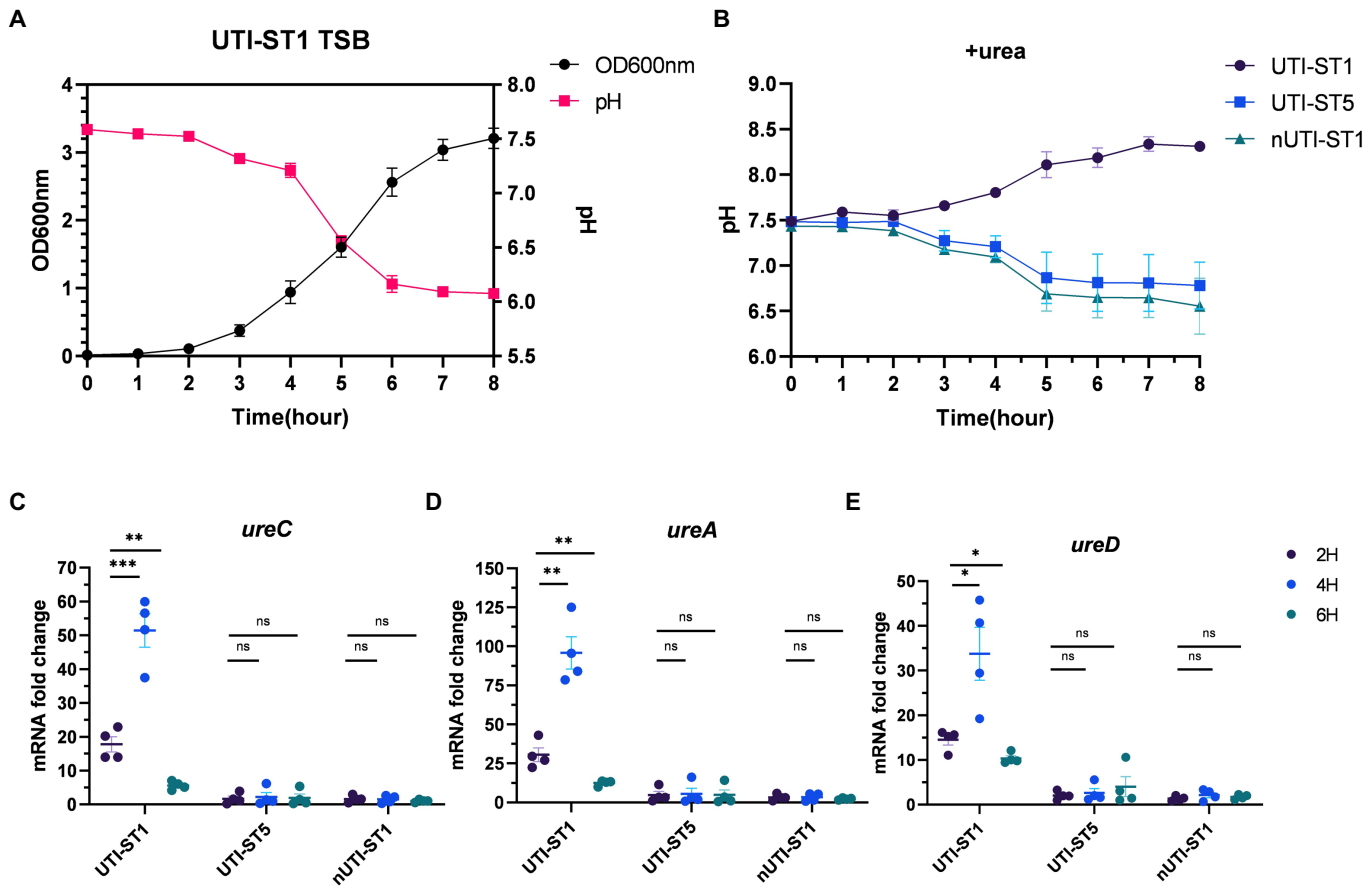
Variations in urease gene expression with the environmental pH. **(A)** The pH and growth curve of urinary tract infection-derived-sequence type (UTI-ST)-1 in tryptic soy broth (TSB) without urea, as determined by optical density measurement at 600 nm; **(B)** the pH curve of UTI-ST1, UTI-ST5, and non-UTI-derived (nUTI)-ST1 in TSB medium supplemented with urea. **(C–E)** The transcription level of *ureACD* in UTI-ST1, UTI-ST5, and nUTI-ST1 at 2, 4, and 6 h, on TSB without urea. Each dot in **(C–E)** is an average of the triplicates of each strain. Unpaired Student’s *t*-test was used in these analyses, ^*^p < 0.05; ^**^p < 0.01; ^***^p < 0.001; ns, not significant.

Previous studies have outlined urease as more than a virulence factor. Urease also acts as an acid resistance regulator to combat the low-pH environment (Cotter and Hill, 2003). With the growth of the bacteria, the pH of the culture drops, due to the accumulation of acid metabolites (Somerville and Proctor, 2009; Bronesky et al., 2016). We monitored the pH of the UTI-ST1 culture in urea-supplemented and unsupplemented TSB media for 8 h (Figures 4A,B) and found that in the absence of urea, the pH of the culture reduced to approximately 6.0 from 7.50 (Figure 4A). In the urea-supplemented media, the pH of the UTI-ST1 culture gradually rose to approximately 8.20 after 8 h, due to the urea hydrolysis caused by UTI-ST1 urease (Figure 4B); however, the pH of the UTI-ST5 and nUTI-ST1 cultures remained low (~6.5; Figure 4B). Thereafter, we monitored the continuous transcription of UTI-ST1 *ureACD* at 2, 4, and 6 h, on TSB without urea. At 4 h, when the culture pH was approximately 7.0, *ureACD* transcription was approximately 2–3 times higher than that at 2 h (pH 7.50). Furthermore, at 6 h, *ureACD* transcription declined 3–10 times compared to that at 4 h, with a drop in the culture pH (~6.0; Figures 4C–E). Meanwhile, both UTI-ST5 and nUTI-ST1 showed low urease gene transcription and low culture pH (Figures 4C–E). These results indicate that the urease gene transcription increases with the drop in pH and peaks in a neutral to mildly acidic environment. When the environmental pH continuously decreases, the transcription of urease would be repressed.

### Agr regulates urease gene expression

**Figure 5:**
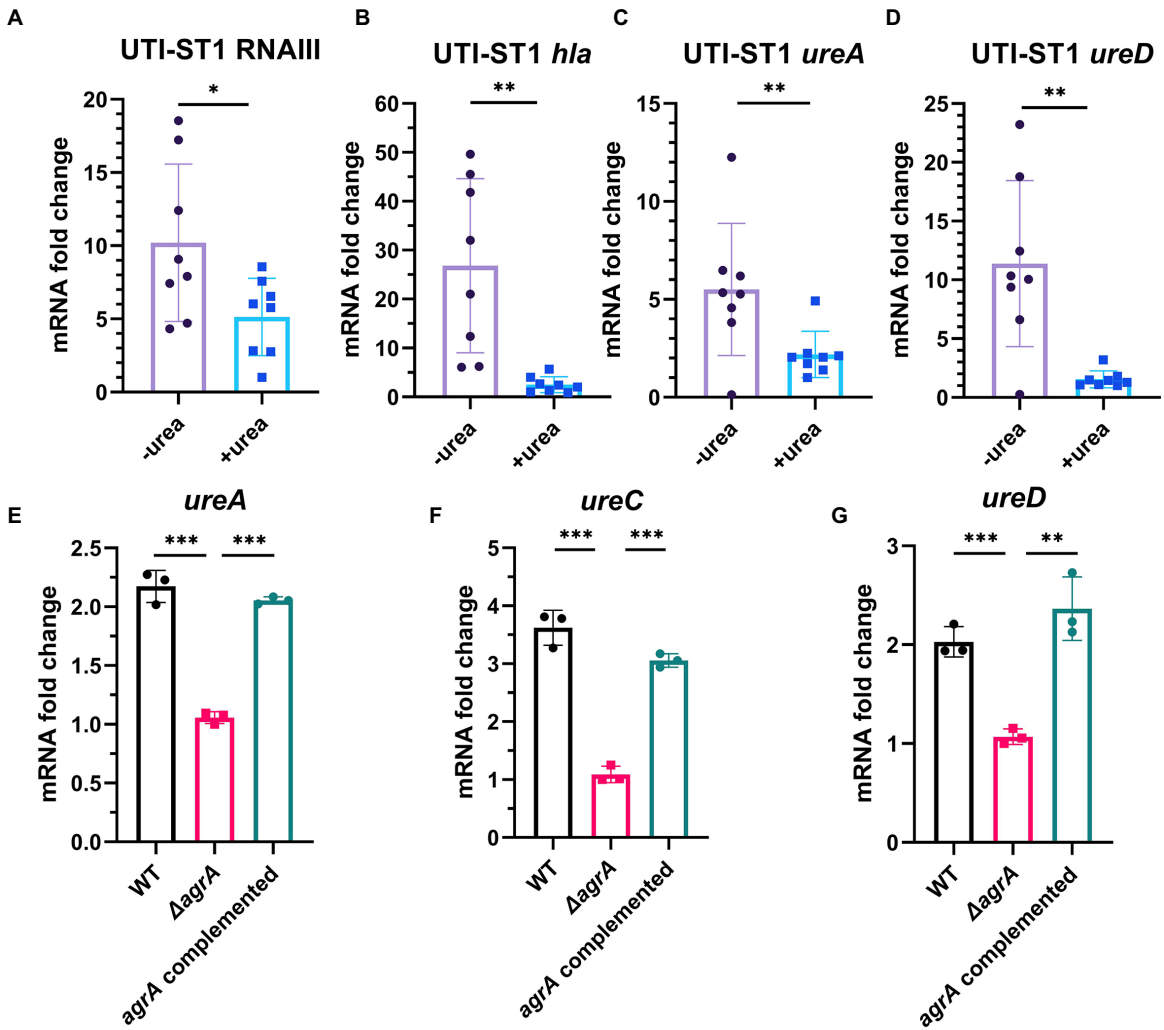
The regulation of urease gene transcription by the Agr system, with the change in environmental pH. **(A–D)** The transcription level of RNAIII (*agr* effector gene), *hla*, and *ureAD* (urease gene) in the presence or absence of urea-supplementation (2%) at 4 h. **(E–G)** The expression of *ureACD* in the urinary tract infection-derived-sequence type (UTI-ST)-1 wildtype (WT) and UTI-ST1 *agrA* mutant. Each dot in **A–D** is an average of the triplicates of each strain. Statistical analyses were performed using unpaired Student’s *t*-test, ^*^p < 0.05; ^**^p < 0.01; ^***^p < 0.001; ns, not significant.

**Figure 6:**
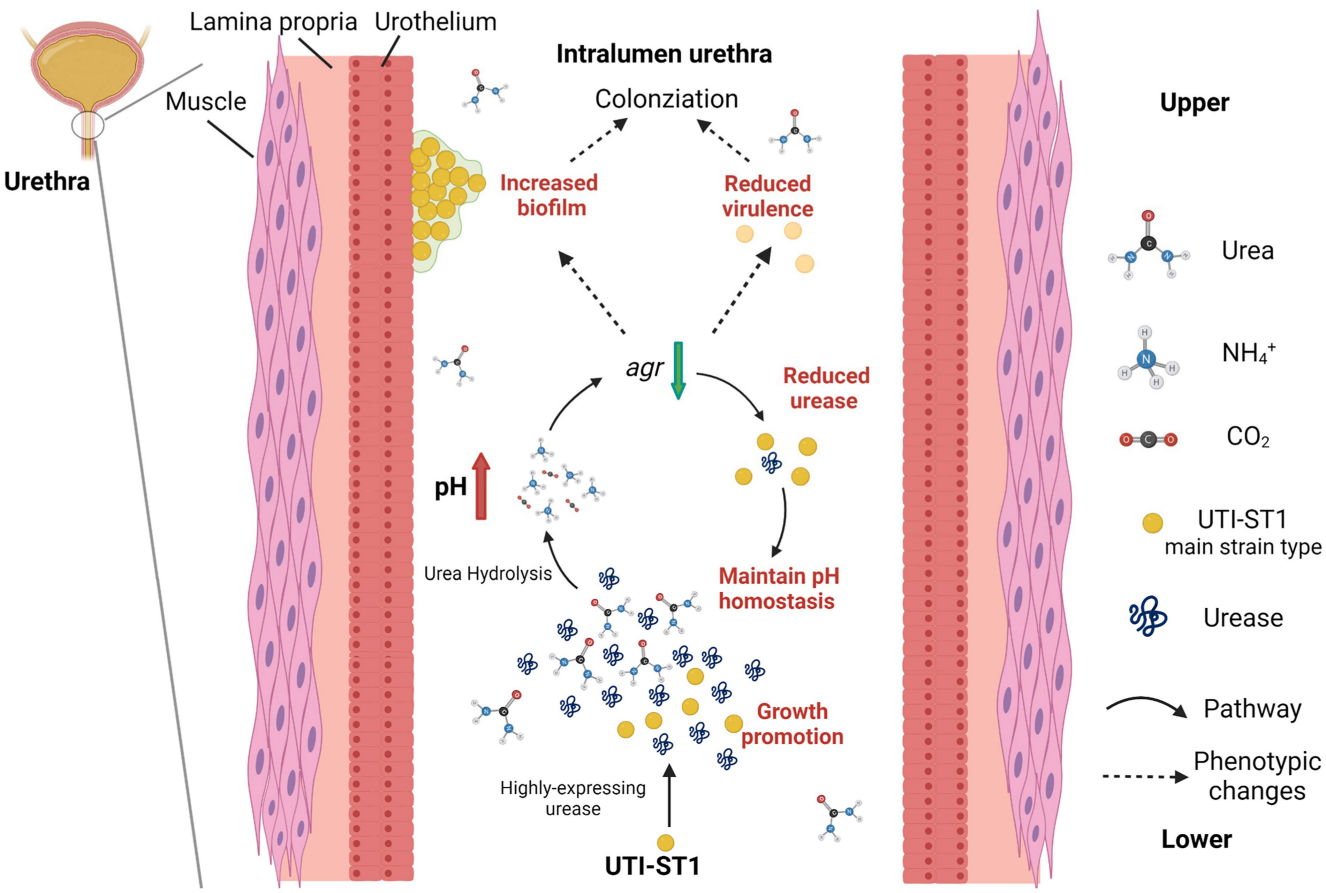
Model of the pathogenesis process in the urinary tract of UTI-ST1 *Staphylococcus aureus* by highly expressing urease. The utilization of urea promoted the growth ability of UTI-ST1 *S. aureus* and the environmental pH was elevated by the hydrolysis products, ammonia. When pH rose to some degree, the urease expression would be down-regulated by the Agr system to maintain a relevant stable pH condition. As a result, the biofilm formation ability was promoted and the virulence was weakened, thus helping the UTI-ST1 to colonize the urinary tract with even persistence.

Urease gene expression is a complex network, and *ccpA* (Zhou et al., 2019), *codY* (Huang et al., 2014), and *agr* (Queck et al., 2008; Bronesky et al., 2016) have been predicted to be involved in the regulation of the urease gene. A study has reported *ccpA* and *codY* as positive and negative regulators of the urease gene expression, respectively (Zhou et al., 2019). In addition, Agr, a critical quorum sensing system in *S. aureus*, has been proven to upregulate urease gene expression; however, its role in urease gene expression during UTI pathogenesis has not yet been determined. The Agr system consists of two adjacent transcripts, RNAII and RNAIII, whose expression is driven by P2 and P3 promoters, respectively. RNAII transcript is an operon of four genes (*agrBDCA*) encoding the machinery of the quorum sensing system, whereas RNAIII transcript encodes the primary effector that regulates the expression of most *agr*-dependent downstream target genes (Peng et al., 1988). Therefore, we evaluated the transcription of Agr system-associated genes and *ureAD*. At 4 h of incubation, the transcription of RNAIII and *hla*, a downstream regulatory virulence gene of the Agr system, was reduced by approximately 2-fold and 10-fold in the urea-supplemented media (pH ~7.7) compared to that in the urea-unsupplemented media (pH ~7.0; Figures 5A,B), indicating that the Agr system was inhibited in the alkaline environment, due to ammonia generation from urea degradation. Meanwhile, at 4 h of incubation, the transcription of *ureA* and *ureD* in the urea-supplemented media (pH ~7) also reduced by approximately 2.5-fold and 7.4-fold, respectively, compared to that in the urea-unsupplemented media (Figures 5C,D). Combined with the phenotypic changes in the urea-supplemented media, the Agr system senses the variations in the environmental pH to adapt to the environmental conditions during the bacterial growth process. The Agr system functions actively under neutral pH conditions and declines under alkaline and acidic conditions (Regassa and Betley, 1992), suggesting that it may downregulate virulence genes and upregulate biofilm-forming genes, during UTI pathogenesis (Queck et al., 2008; Arciola et al., 2012). Finally, we constructed UTI-ST1 *agrA* mutant and complemented strains and found that the *ureACD* expression of the mutant was significantly declined compared to that of the UTI-ST1 WT (Figures 5E–G), suggesting that *agr* plays a role in upregulating the urease genes, which might help in maintaining the optimal environmental pH (Figure 6).

## Discussion

An overarching objective of our research is to delineate the molecular epidemiology, phenotypic characteristics, and pathophysiology of *S. aureus*-induced UTIs. In this study, we collected 4,405 *S. aureus* isolates from various clinical sources during 2008–2020 from a general Hospital in Shanghai (China) and examined their STs, virulence characteristics, and antibiotic resistance. In this study, we also analyzed the role of urease in UTI-ST1-induced pathogenesis, since UTI-ST1 was one of the main STs of UTI-SA isolates. The antibiotic resistance profile revealed that most UTI-ST1 and UTI-ST5 isolates were MRSA strains, which can cause difficulties in clinical therapy. While UTI-ST1 isolates showed high resistance to levofloxacin (Quinolones), UTI-ST5 isolates showed resistance to aminoglycoside and macrolide antibiotics (Table 2). In a previous study, Cortes et al. analyzed the antimicrobial resistance traits of ST1 MRSA and found ST1 MRSA strains might be highly resistant to aminoglycosides and probably gained intrinsic ciprofloxacin resistance for amino acid substitution (Côrtes et al., 2021). While, the common HA-MRSA ST5 isolates were found to harbor a large number of resistance genes (Jian et al., 2021), which correlates with the antimicrobial profile in our study. Compared to the UTI-ST5 and nUTI-ST1 isolates, UTI-ST1 displayed declined hemolytic ability and increased biofilm formation and adhesion ability under urea supplementation. In addition, UTI-ST1 showed active urease gene expression and urease activity, while the other sequence types such as UTI-ST5, UTI-ST7, UTI-ST398, and nUTI-SA did not display a similar trend. Moreover, the UTI-ST1 *ureC* mutant displayed similar hemolytic ability and biofilm-forming phenotypes under urea-supplementation, and the urine CFU enumeration of the mutant reduced rapidly in the murine UTI model, while UTI-ST1 could not be eliminated so quickly. In addition, we found that UTI-ST1 phenotype and urease expression were regulated by the environmental pH and potentially regulated by the Agr system. Urea is the only substrate to urease and it is neutral itself, which means it may not impact the growth and pathogenesis of urease-negative bacteria. However, the urease-positive ones can hydrolyze the urea to produce ammonia, an alkaline product, to survive and persist in an acidic environment. With the change in pH, the Agr system regulates the expression of hemolysis, urease, and many other genes, resulting in phenotype changes (Figure 6). Moreover, our results highlight the role of urease, as a urea environment regulator, boosting *S. aureus* persistence in the nutrient-limiting urinary niche.

Chronic UTI is a persistent and frequent issue, especially in the elderly, female, and catheter-implanted patients (Flores-Mireles et al., 2015; Kitano et al., 2021). It is widely accepted that the female gender is a risk factor for UTIs (Foxman, 2010); however, the majority of UTI-ST1 (65.85%) and UTI-ST5 (81.67%) isolates were recovered from men, while 34.15% and 18.33% were recovered from women (Supplementary Figure S1), consistent with the previous reports that men are more susceptible to *S. aureus* infections (Gu et al., 2016; Dai et al., 2019; Thomsen et al., 2019). In this study, we found two common UTI-associated STs of *S. aureus* ST1 and ST5. Furthermore, by using a modified semi-quantitative urease detection assay, we discovered that the UTI-ST1 isolates displayed significantly higher urease activities than the other STs. Moreover, UTI-ST1 was found to degrade urea to generate ammonia, which promotes its growth and persistence by increasing its biofilm-forming ability and adhesion to the epithelium. Although UTI-ST5 did not show obvious urease activity, it displayed a strong biofilm-forming ability and mild virulence, which might facilitate initiating infection. As a conserved virulence factor, urease is widely present in plants, fungi, bacteria, and other organisms (Dixon et al., 1975; Rutherford, 2014; Gu et al., 2016). In previous studies, Gram-negative bacteria were the hotspots for UTI research (Nielubowicz and Mobley, 2010; Pitout, 2012; Foxman, 2014; Schaffer and Pearson, 2015; Clegg and Murphy, 2016; Terlizzi et al., 2017; Klingeberg et al., 2018). Therefore, *S. aureus*, with a relatively simple cell structure compared to the Gram-negative bacteria, was not considered a common cause of UTI. Fimbriae, pili, and secreted toxins are important virulence factors of uropathogenic *E. coli* (Terlizzi et al., 2017), and urease is an important virulence factor in *Proteus mirabilis*, which is associated with renal stone formation (Schaffer and Pearson, 2015; Clegg and Murphy, 2016). Owing to the frequent flush movement of the urine, the lack of nutrients, pH variations, etc., the urinary tract is a difficult environment for pathogens to colonize. However, the urease detection method is currently not common in routine clinical practice. In recent years, the ^13^C-urea breath test and the rapid urease test have been widely applied to detect *H. pylori* in the stomach (Ricci et al., 2007). In this study, we applied a modified semi-quantitative urease test (Duran Ramirez et al., 2022), which can be used to detect urease-producing bacteria. Both Gram-negative and Gram-positive urease-producing bacteria can hydrolyze urea to facilitate growth and regulate the environmental pH (Cotter and Hill, 2003). However, pH plays a complicated role in promoting renal stone formation (Henderson, 1993; Bihl and Meyers, 2001). Previous studies found that low urine pH may cause the deposition of calcium oxalate stones, while >7.0 pH of urine is found in struvite or triple phosphate stones, caused by *Proteus* and *Klebsiella* infections (Bihl and Meyers, 2001). However, a few studies hypothesized elevated pH as a lithogenic risk factor (Henderson, 1993; Schaffer et al., 2016). Different renal stone components are deposited in different pH environments. For those undergoing chronic UTI, the increased pH of urine can be a lithogenic risk factor for urease-positive bacteria and can be monitored.

*Staphylococcus aureus* contains various virulence genes and corresponding regulatory systems, with the Agr quorum sensing system being the most important (Regassa and Betley, 1992; Bronesky et al., 2016; Matsumoto et al., 2021). It is predicted and proven that urease is directly upregulated by the Agr system in an RNAIII-dependent manner (Queck et al., 2008; Arciola et al., 2012), which correlates with the RT-qPCR results (Figures 4C–E). In our study, we deeply studied this relationship combined with the phenotypic changes in urea-supplied medium and the potential pathogenesis of UTI-ST1. The variations in *agr* expression under different pH environments (Regassa and Betley, 1992) confirm the weakened virulence phenotype and enhanced biofilm-forming and adhesion ability of the UTI-ST1. However, we failed to observe the increased transcription of the biofilm regulator gene, *ica* (Kırmusaoğlu and Kaşıkçı, 2020). This could be because the biofilm is controlled by multiple genes, such as *sarH1* (Queck et al., 2008), *sasG* (Geoghegan et al., 2010), and several *ica*-independent pathways. It has been proved that *agr* mutant is susceptible to evading the cytotoxic effects of neutrophils (Zhou et al., 2019; Matsumoto et al., 2021), which was also observed in the urea-supplemented and unsupplemented UTI-ST1 groups (Supplementary Figure S5); however, the underlying mechanism is yet to be revealed. In previous studies, the expression of urease genes was found to be induced by a mildly acid environment (Zhou et al., 2019). However, the transcription of urease gens dropped at pH 6 in our study (Figures 4C–E). It might be because we applied a different method to demonstrate the expression of urease genes. We directly detect the RNA level by RT-PCR while the researchers quantified the urease expression in translation level in previous studies, which might include additive effects. In addition, the change of mRNA is usually prior to protein expression so that our opinions did not conflict with others.

In conclusion, our data indicate that UTI-ST1 is the main strain type of UTI-SA and that UTI-ST1 highly expresses urease, which hydrolyzes urea to produce ammonia and elevate the environmental pH. The alkaline environment further declines the expression of the Agr system, resulting in weakened virulence and improved biofilm and adhesion of UTI-ST1. Therefore, our findings demonstrate the importance of urease in the survival and persistence of ST1 *S. aureus* in the urinary tract during UTI pathogenesis.

## Data availability statement

The original contributions presented in the study are included in the article/Supplementary material, further inquiries can be directed to the corresponding authors.

## Ethics statement

The animal study was reviewed and approved by the Ethics Committee of Renji Hospital, School of Medicine, Shanghai Jiaotong University, Shanghai, China.

## Author contributions

LH and ML: conceptualization, funding acquisition, supervision, and writing—review and editing. KX, LH, and ML: methodology. LH, KX, YW, TC, and YJ: investigation. KX, LH, ML, QL, and HW: visualization. KX: writing—original draft. All authors contributed to the article and approved the submitted version.

## Funding

This study was supported by the National Natural Science Foundation of China [grant numbers 82272395 (to LH), 81974311 (to LH), 81873957 (to ML), 82172325 (to ML), and 82102455 (to YW)], the Shanghai Pujiang Program [grant number 2019PJD026 (to LH)], and the Shanghai Sailing Program [21YF1425500 (to YW)].

## Conflict of interest

The authors declare that the research was conducted in the absence of any commercial or financial relationships that could be construed as a potential conflict of interest.

## Publisher’s note

All claims expressed in this article are solely those of the authors and do not necessarily represent those of their affiliated organizations, or those of the publisher, the editors and the reviewers. Any product that may be evaluated in this article, or claim that may be made by its manufacturer, is not guaranteed or endorsed by the publisher.

## Footnotes

^1^ http://www.pubmlst.net
