# Supplementary material for: Staphylococcus aureus ST1 promotes persistent urinary tract infection by highly expressing the urease
Source: Front Microbiol. 2023 Feb 22;14:1101754. doi: 10.3389/fmicb.2023.1101754 (PMC9992547; doi:10.3389/fmicb.2023.1101754)
Supplement: Supplementary file 1 [file Data_Sheet_1.zip › Supplementary Figures.docx]

Supplementary Material

## Supplementary Figures

##
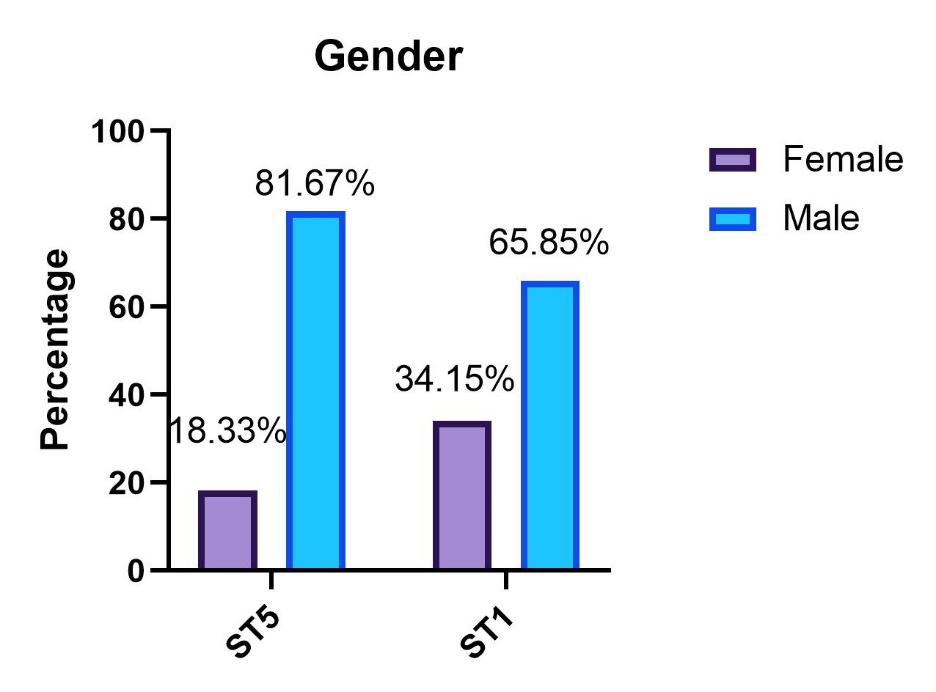


**Figure S1**. The gender distribution of UTI-ST1 and UTI-ST5.


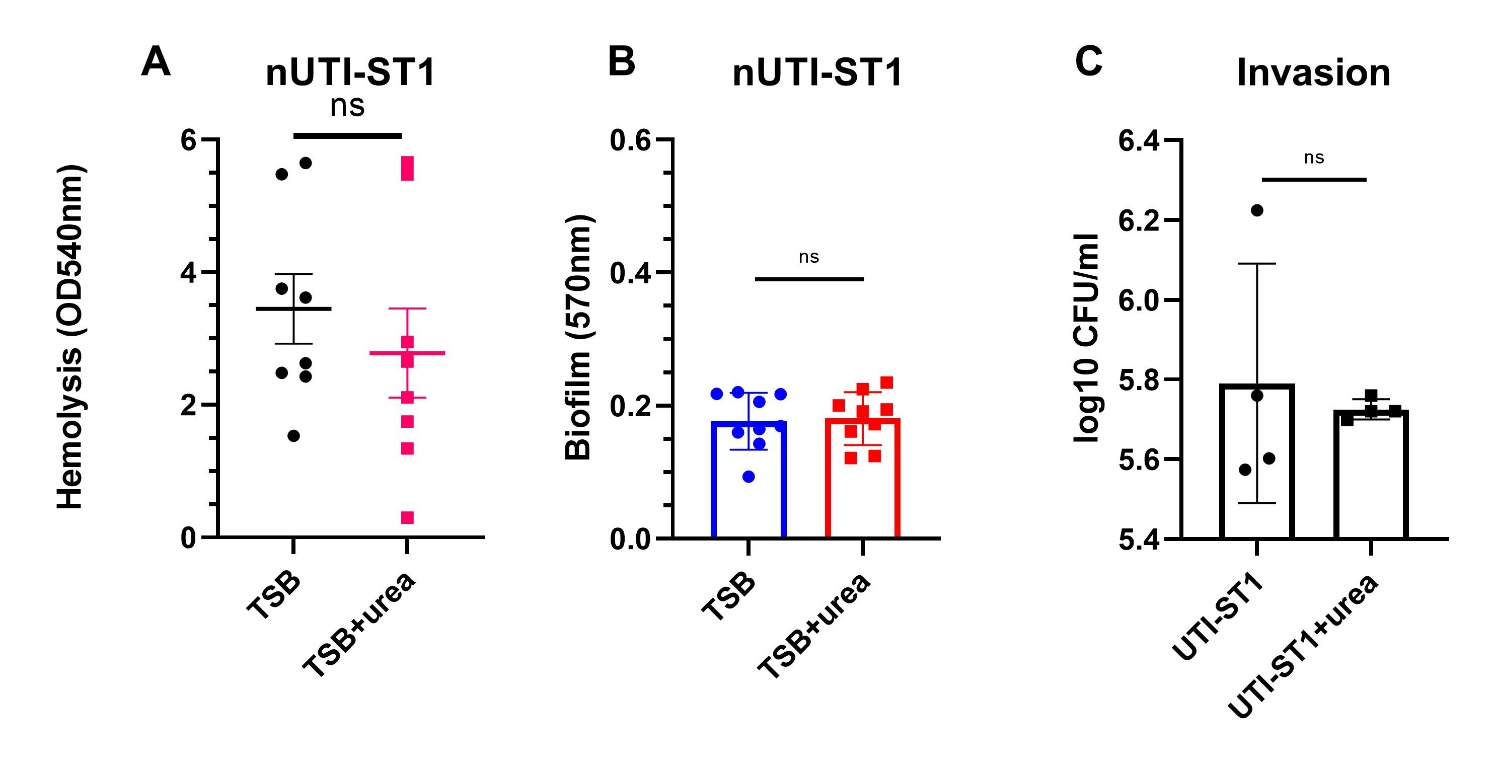


**Figure S2**. Hemolysis and biofilm formation and invasion of bladder epithelium 5637 of non-UTI derived ST1 or UTI-ST1 isolations: A) hemolysis test of nUTI-ST1 with or without urea; B) biofilm formation assay of nUTI-ST1 with or without urea; C) invasion assay of bladder epithelium 5637 of UTI-ST1 with or without urea. Each dot in A-C is an average of the triplicates of each strain. Statistical significance was assessed using unpaired two-tailed Student’s t-tests; ns, no significant.

**
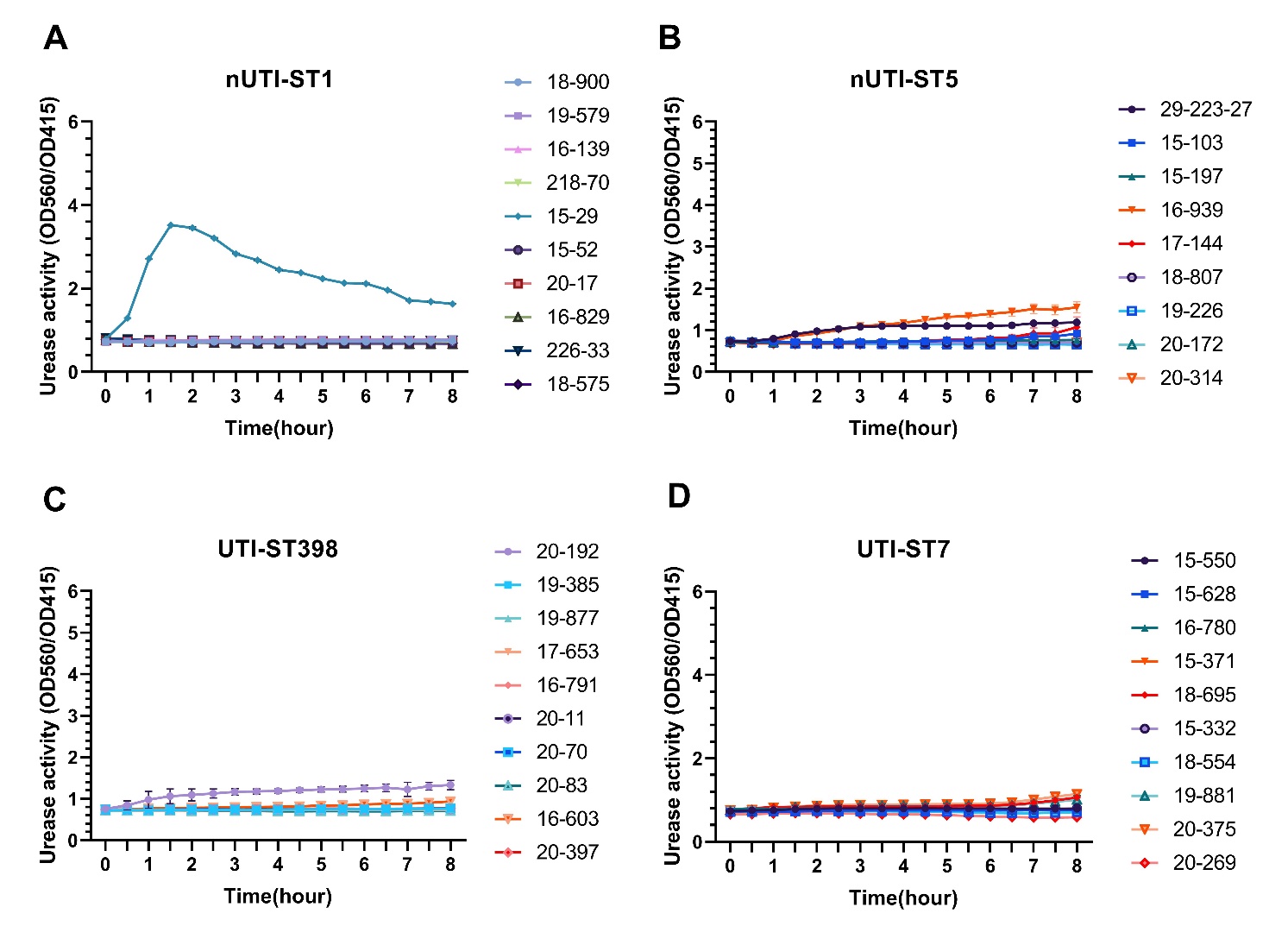
**

**Figure S3**. Urease activity of UTI or nUTI-derived strains: nUTI-ST1 (A), nUTI-ST5 (B), UTI-ST398 (C) and UTI-ST7 (D). 10 isolations were randomly selected from each sequence type respectively. The data of OD560nm and OD415nm were collected continuously at 37℃ cultivation.


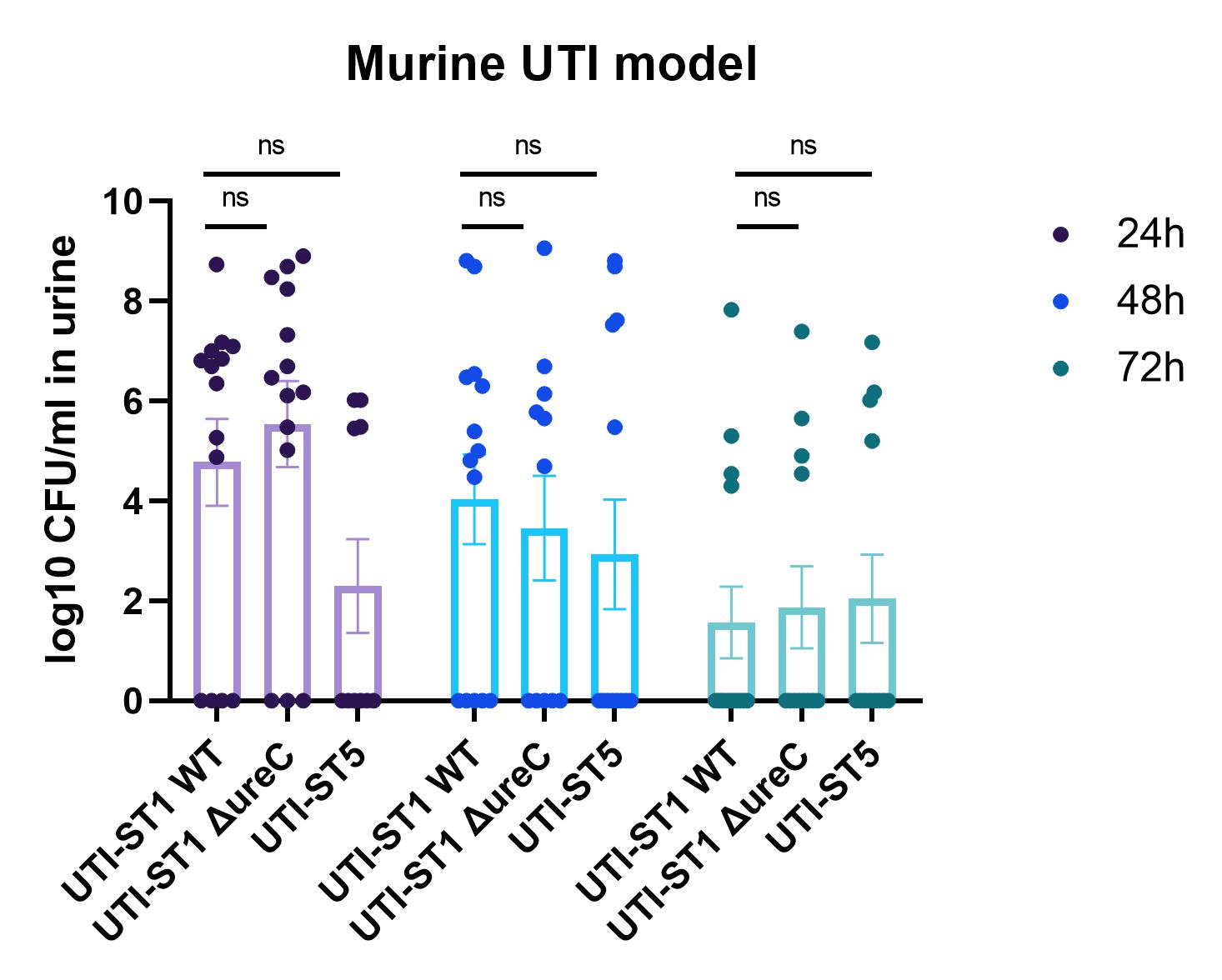


**Figure S4.** The urine CFU enumeration at 24, 48 and 72hpi of UTI-ST1 WT, UTI-ST1 *ureC* mutant and UTI-ST5 in murine UTI model. Statistical analyses were performed using unpaired Student’s t-test, **p* <0.05; ***p* <0.01; ns, not significant.

**
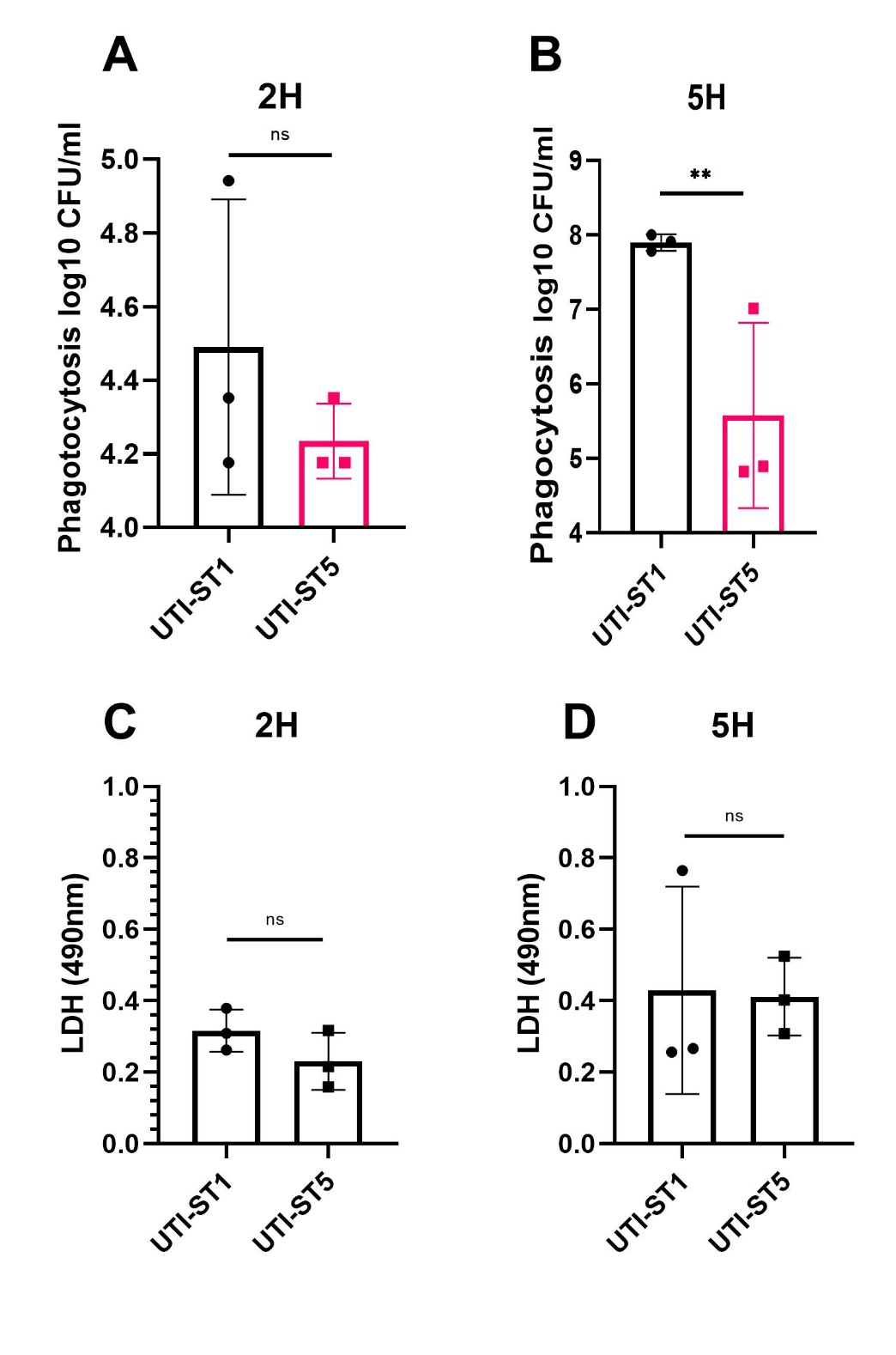
**

**Figure S5**. The neutrophils phagocytosis and lactate dehydrogenase test (LDH) of UTI-ST1 and UTI-ST5. (A-B) Neutrophils were separated from human blood (EDTA-anticoagulant) by density gradient centrifugation. UTI-ST1 and UTI-ST5 isolations were incubated with neutrophils at 1:100 for 2h or 5h in 96-well plate. Although the CFU/ml of UTI-ST1 and UTI-ST5 was similar at 2h (A), at 5h, the alive bacteria of UTI-ST1 remaining in neutrophils was significantly higher than UTI-ST5 (B). Every result was carried out in triplicates. (C-D) The lactate dehydrogenase in the supernatant was detected by cytotoxity detection kit (LDH) (Roche) and there was no difference between UTI-ST1 and UTI-ST5 in cytotoxity. Each dot in A-D is an average of the triplicates of each strain.

**Supplementary Tables**

**Table S1. UTI-ST1 *ureC* and *agrA* mutant construction**

| **Number** | **Gene** | **Sequence** | **Length (bp)** |
| --- | --- | --- | --- |
| 1 | *ureC*-A | TTATCCACTTCCAATGTCATTAATATACTACTATTC | 36 |
| 2 | *ureC*-B | CACCGTTATCTCCGAATACA | 20 |
| 3 | *ureC*-C | TGTATTCGGAGATAACGGTGTTGCCAATTTGTCAAATTCA | 40 |
| 4 | *ureC*-D | TACTTCCAATCCAATGCAAGTGGAATAGCACGAACA | 36 |
| 5 | *agrA*-A | TTATCCACTTCCAATGTGTAAGATTGCCATTATGGG | 36 |
| 6 | *agrA*-B | AGTCAGTTAACGGCGTATTC | 20 |
| 7 | *agrA*-C | GAATACGCCGTTAACTGACTGATCGTCTTCGCAAATGAAA | 40 |
| 8 | *agrA*-D | GAATACGCCGTTAACTGACTGATCGTCTTCGCAAATGAAA | 36 |
| 9 | *ureC* SmaI-F | GAGCCCGGGTAAGTTAATCACAGTACATC | 29 |
| 10 | *ureC*-BamHI-R | GCCGGATCCAATTGATGGGCTATATCTC | 28 |
| 11 | *agrA*-SmaI-F | GAGCCCGGGGAAGACGATCCAAAACAAAG | 29 |
| 12 | *agrA*-BamHI-R | GCCGGATCCTTAACTGACTTTATTATCTT | 29 |

**Table S2. The basic information of the strains applied in vitro experiments**

| **Number** | **Name** | **Source** | **Patient's gender** | **Patient's age** | **SPA type** | **MLST type** |
| --- | --- | --- | --- | --- | --- | --- |
| 1 | 15-0068 | urine | m | 87 | t321 | ST1 |
| 2 | 15-0092 | urine | f | 87 | t321 | ST1 |
| 3 | 15-0144 | urine | f | 85 | t321 | ST1 |
| 4 | 15-0163 | urine | f | 87 | t321 | ST1 |
| 5 | 15-0215 | urine | m | 77 | t321 | ST1 |
| 6 | 15-0306 | urine | m | 75 | t321 | ST1 |
| 7 | 15-0452 | urine | f | 79 | t321 | ST1 |
| 8 | 15-0200 | urine | m | 87 | t321 | ST1 |
| 9 | 12-0771 | urine | f | 89 | t321 | ST1 |
| 10 | 18-0879 | urine | m | 73 | t127 | ST1 |
| 11 | 20-0108 | urine | f | 73 | t548 | ST5 |
| 12 | 19-0410 | urine | m | 79 | t002 | ST5 |
| 13 | 19-0728 | urine | m | 79 | t002 | ST5 |
| 14 | 18-0688 | urine | m | 95 | t002 | ST5 |
| 15 | 18-0691 | urine | f | 96 | t002 | ST5 |
| 16 | 17-0570 | urine | m | 39 | t311 | ST5 |
| 17 | 17-0790 | urine | m | 62 | t002 | ST5 |
| 18 | 16-0477 | urine | f | 63 | t002 | ST5 |
| 19 | 15-0111 | urine | m | 83 | t002 | ST5 |
| 20 | 29-223-36 | urine | m | 81 | t002 | ST5 |
| 21 | 20-0192 | urine | m | 53 | t034 | ST398 |
| 22 | 19-0385 | urine | f | 59 | t8100 | ST398 |
| 23 | 19-0877 | urine | m | 53 | t034 | ST398 |
| 24 | 17-0653 | urine | f | 51 | t034 | ST398 |
| 25 | 16-0791 | urine | m | 70 | t034 | ST398 |
| 26 | 20-0011 | urine | m | 53 | t034 | ST398 |
| 27 | 20-0070 | urine | f | 66 | t034 | ST398 |
| 28 | 20-0083 | urine | m | 15 | t034 | ST398 |
| 29 | 16-0603 | urine | f | 57 | t2383 | ST398 |
| 30 | 20-0397 | urine | f | 64 | t034 | ST398 |
| 31 | 15-0550 | urine | m | 53 | t803 | ST7 |
| 32 | 15-0628 | urine | m | 34 | t1361 | ST7 |
| 33 | 16-0780 | urine | f | 31 | t605 | ST7 |
| 34 | 15-0371 | urine | m | 77 | t2932 | ST7 |
| 35 | 18-0695 | urine | f | 91 | t091 | ST7 |
| 36 | 15-0332 | urine | m | 23 | t796 | ST7 |
| 37 | 18-0554 | urine | m | 91 | t091 | ST7 |
| 38 | 19-0881 | urine | f | 17 | t091 | ST7 |
| 39 | 20-0375 | urine | f | 70 | t091 | ST7 |
| 40 | 20-0269 | urine | m | 63 | t3932 | ST7 |
| 41 | 18-0900 | blood | m | 95 | t321 | ST1 |
| 42 | 19-0579 | sputum | m | 97 | t127 | ST1 |
| 43 | 16-0139 | sputum | f | 86 | t321 | ST1 |
| 44 | 218-70 | sputum | m | 63 | t127 | ST1 |
| 45 | 15-0029 | sputum | m | 87 | t321 | ST1 |
| 46 | 15-0052 | sputum | m | 88 | t321 | ST1 |
| 47 | 20-0017 | sputum | m | 90 | t127 | ST1 |
| 48 | 16-0829 | sputum | m | 85 | t321 | ST1 |
| 49 | 226-33 | wound | m | 72 | t127 | ST1 |
| 50 | 18-0575 | sputum | m | 90 | t127 | ST1 |
| 51 | 29-223-27 | sputum | m | 81 | t002 | ST5 |
| 52 | 15-0103 | sputum | m | 83 | t002 | ST5 |
| 53 | 15-0197 | sputum | m | 84 | t311 | ST5 |
| 54 | 16-0939 | skin | m | 14 | t2460 | ST5 |
| 55 | 17-0144 | sputum | m | 68 | t002 | ST5 |
| 56 | 18-0807 | sputum | m | 75 | t2460 | ST5 |
| 57 | 19-0226 | sputum | m | 86 | t002 | ST5 |
| 58 | 20-0172 | sputum | m | 46 | t311 | ST5 |
| 59 | 20-0314 | skin | m | 23 | t062 | ST5 |
